# A SLC6 transporter cloned from the lion's mane jellyfish (Cnidaria, Scyphozoa) is expressed in neurons

**DOI:** 10.1371/journal.pone.0218806

**Published:** 2019-06-24

**Authors:** Christelle Bouchard, Dmitri Y. Boudko, Rays H. Y. Jiang

**Affiliations:** 1 College of Science and Mathematics, University of South Florida, Sarasota, Florida, United States of America; 2 Department of Physiology and Biophysics, Rosalind Franklin University of Medicine and Science, Chicago Medical School, North Chicago, Illinois, United States of America; 3 Global and Planetary Health, College of Public Health, University of South Florida USF Genomics Program, Tampa, Florida, United States of America; Nanjing University, CHINA

## Abstract

In the course of recent comparative genomic studies conducted on nervous systems across the phylogeny, current thinking is leaning in favor of more heterogeneity among nervous systems than what was initially expected. The isolation and characterization of molecular components that constitute the cnidarian neuron is not only of interest to the physiologist but also, on a larger scale, to those who study the evolution of nervous systems. Understanding the function of those ancient neurons involves the identification of neurotransmitters and their precursors, the description of nutrients used by neurons for metabolic purposes and the identification of integral membrane proteins that bind to those compounds. Using a molecular cloning strategy targeting membrane proteins that are known to be present in all forms of life, we isolated a member of the solute carrier family 6 from the scyphozoan jellyfish *Cyanea capillata*. The phylogenetic analysis suggested that the new transporter sequence belongs to an ancestral group of the nutrient amino acid transporter subfamily and is part of a cluster of cnidarian sequences which may translocate the same substrate. We found that the jellyfish transporter is expressed in neurons of the motor nerve net of the animal. To this end, we established an *in situ* hybridization protocol for the tissues of *C*. *capillata* and developed a specific antibody to the jellyfish transporter. Finally, we showed that the gene that codes for the jellyfish transporter also expresses a long non-coding RNA. We hope that this research will contribute to studies that seek to understand what constitutes a neuron in species that belong to an ancient phylum.

## Introduction

Modern day cnidarians are descendant of ancient metazoan lineages. They share a common ancestor with bilateral organisms, and additionally, possess neuronal systems with evident synaptic ultrastructures [1]. Their study provides a unique opportunity to gain a better understanding of the early evolutionary stages of cellular, metabolic, and neurochemical integration in animals. The annotation of the starlet sea anemone [2] and Hydra genomes [3] revealed that cnidarians possess many orthologous genes present in the nervous system of the most advanced bilateral organisms [4, 5]. Nonetheless, the identity of neurotransmitters in cnidarians remains enigmatic. This may be due to the fact that our search for elements involved in chemical neurotransmission has been based on the premise of a monophyletic origin of nervous systems, a notion that has been recently challenged [6]. In the case of the polyphyletic hypothesis, wherein nervous systems evolved more than once in different lineages, one would expect to observe a diversity of neuronal phenotypes. Alternatively, there could still be significant differences between nervous systems even if these systems emerged from a single original source. On this last point, divergent evolutionary histories of a primordial nervous system could also be conceived as the causal factor behind the heterogeneity of nervous systems and neuronal networks [7]. In both cases, the identity of molecular components underlying neuronal communication would vary greatly between animals of ancient and recent phyla.

Membrane transporters form a Major Facilitator Superfamily (MFS) of transmembrane proteins which couple translocation of substrates and key electrolytes [8]. Because transmembrane fluxes of substrates and electrolytes are essential to life, membrane transporters likely arose with the first cell, which may explain why the principal structural motifs present in transporters of bacteria remain conserved in those of metazoans [9, 10]. On the other hand, metazoan evolution triggered an expansion of transporter diversity necessary to handle cellular competition for nutrients in tandem with escalation of cellular complexity.

Some key phenomena of metazoan evolution such as acquisition of neuronal integration and massive extinction of enzymatic pathways involved in the synthesis of the most energetically-expensive amino acids, required a dramatic expansion of transporter diversity in order to distribute the “now essential” proteinogenic substrates along with the membrane translocation of metabolites and neurotransmitters derived from these substrates [11, 12]. The Sodium Neurotransmitter symporter Family (SNF) a.k.a. SoLute Carrier family 6 (SLC6) represents a striking example of such an expansion [13, 14]. It includes the repertoire of transporters for essential and conditionally essential amino acids, their metabolic derivatives, as well as amino acid neurotransmitters GABA, glycine and glutamate; and derivatives of essential amino acids phenylalanine and tryptophan which include the monoamines serotonin, dopamine, octopamine, epinephrine and norepinephrine.

Intriguingly, phylogenomic trees of members of the SLC6 family show distinct clusters for amino acid-selective and neurotransmitter-selective transport mechanisms. The separation of a neurotransmitter transporter cluster from SLC6 amino acid transporter groups, which occurred early during metazoan evolution, was essential for metabolic integration of metazoan animals [10, 12, 13]. In order to shed light on metabolic requirements of earlier neurons, we searched for membrane protein targets likely to be conserved that yet remain unexplored in cnidarians, and that may ultimately provide more information on neuronal function. Here we report the molecular cloning and the localization of the first neuronal SLC6 transporter from the Lion's mane jellyfish, *Cyanea capillata* (Linnaeus, 1758).

## Material and methods

### Experimental organism

The jellyfish *Cyanea capillata* were acquired from the Marine Biological Laboratory (Woods Hole, MA), and were kept in a jellyfish kreisel supplied with filtered sea water from the Atlantic. The water was chilled at 15°C and aerated. Animals were fed mud minnows collected with a cast net from the intra-coastal waterway near the shore land of the University of Florida. No specific permissions were required for using mud minnows (a non-endangered species) at the time of the study.

### Cloning of the *Cyanea* sequence

Degenerate oligonucleotides primers were deduced from conserved regions of annotated SLC6 members. A fragment of 1500 bp was amplified by PCR from 1 μl of a *C*. *capillata* λgt22 phage library [15] using LA Taq DNA polymerase (Takara Bio USA, Inc.) along with a degenerate oligonucleotide (iNAT6R: GCACAACCGGTCCACTCCRTANANCCA) and an oligonucleotide specific to the phage arm (PA144: GTATCGGCGGAATTCGTCGA). Annealing temperature for the PCR amplification was set at 57°C. The fragment was ligated into the pGEM-T vector (Promega, WI, USA) and sequenced. BLAST query revealed that the fragment shared homology with transporters of the SLC6 family. The 5’ and 3’regions were then PCR amplified with oligonucleotides specific to the 1500 bp-fragment of the transporter cDNA (TR-1: CTGATTGCCTTGTTCCAGGT and TR-2: GGGCTGGGTGGATTTCCTTC) paired with primers specific to the respective phage arms (PA143 see sequence above, and PA144: GTATCGGCGGAATTCGTCGA). The full-length fragment was obtained from the same phage library using primers specific to the 5’ and 3’ extremities of the coding sequence of the *C*. *capillata* transporter (TRPX-HindIII: GGGGAAGCTTGCCACCATGCCTGAAGAAATGATAGAAATGGA and TRPX-NotI: GGGGGCGGCCGCTCATCAGGCCATCTTGGATTTAGAGTCATT). A Kozak sequence and restriction sites compatible for directional cloning in the pXOOM vector were added to the primers. This construct was used for heterologous gene expression in HEK-293 cell as well as for cRNA synthesis for expression in *Xenopus laevis* oocytes.

### Phylogenetic analysis

**Fig 1:**
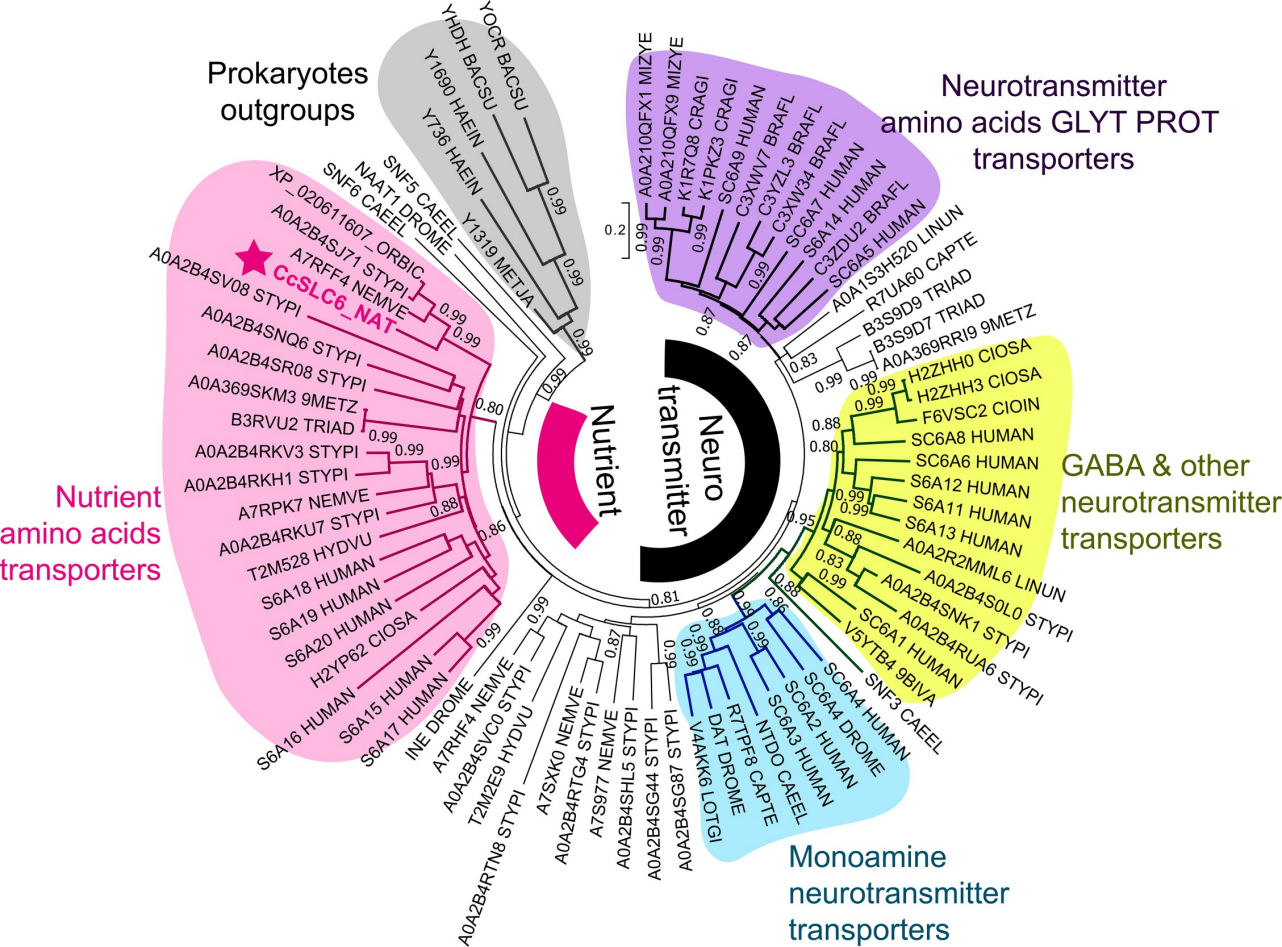
Evolutionary relationships of the newly identified *Cc*SLC6_NAT1 protein with 78 selected SLC6 transporters. The tree shows the major subfamilies of the SLC6 family: The NeuroTransmitter-specific Transporters (NTT) subfamilies and the Nutrient Amino acid (NAT) subfamily. The *C*. *capillata* SLC6 transporter belongs to the NAT subfamily and forms a cluster with related taxa including *Nematostella* representing the closest homologs of the cloned jellyfish transporter. The cnidarian transporters cluster together with characterized B^0^ and IMINO transporters of the NAT subfamily. Other shaded clusters indicate GABAT (GABA transporters), DAT (dopamine transporters) and SERT (serotonin transporters) of the NTT subfamily. Species abbreviation included in leave strings are: BACSU: Bacillus subtilis (strain 168); BIVA: *Bathymodiolus septemdierum*; BRAFL: *Branchiostoma floridae*; CAEEL: *Caenorhabditis elegans*; CAPTE: *Capitella teleta*; CIOIN: *Ciona intestinalis*; CIOSA: *Ciona savignyi*; CRAGI: *Crassostrea gigas*; DROME: *Drosophila melanogaster*; HAEIN: *Haemophilus influenzae* (ATCC 51907); HUMAN: *Homo sapiens*; HYDVU: *Hydra vulgaris*; LINUN: *Lingula unguis*; LOTGI: *Lottia gigantea*; METJA: *Methanocaldococcus jannaschii* (ATCC 43067); MIZYE: *Mizuhopecten yessoensis*; NEMVE: *Nematostella vectensis*; STYPI: *Stylophora pistillata*; ORBIC: *Orbicella faveolata*; TRIAD: *Trichoplax adhaerens*; 9METZ: *Trichoplax* sp. H2. The transporters from prokaryotes were used as an outgroup in the tree. Numbers shown next to the nodes are local support values represented as proportions. UniProt IDs of protein sequences used to assemble this figure are indicated at the ends of branches. Scale bar represents the number of substitutions per site.

The protein sequences of selected SLC6 transporters were retrieved from a BLASTp query of the NCBI’s non-redundant protein sequence database using the translated open reading frame of the cloned *C*. *capillata* transporter. Selected sequences were from the following organisms of specific taxids: *Homo sapiens* (taxid:9606); *Hydra vulgaris* (taxid:6087); *Stylophora pistillata* (taxid:50429); *Bathymodiolus septemdierum* (taxid:220392); *Caenorhabditis elegans* (taxid:6239); *Drosophila melanogaster* (taxid:7227); *Nematostella vectensis* (taxid:45351); *Orbicella faveolata* (taxid:48498); *Trichoplax* sp. H2 (taxid:287889); *Trichoplax adhaerens* (taxid:10228); *Ciona savignyi* (taxid:51511); *Ciona intestinalis* (taxid:7719); *Crassostrea gigas* (taxid:29159); *Lingula unguis* (taxid:7574); *Mizuhopecten yessoensis* (taxid:6573); *Capitella telet*a (taxid:283909); *Lottia gigantea* (taxid:225164); *Haemophilus influenzae* (strain ATCC 51907 / DSM 11121 / KW20 / Rd) (taxid:71421); *Bacillus subtilis* (strain 168) (taxid:224308); *Methanocaldococcus jannaschii* (strain ATCC 43067) (taxid:243232); and *Branchiostoma floridae* (taxid:7739). The taxa were selected to represent deuterostome and protostome taxa, cnidarian groups and other ancestral taxa. The identified proteins were aligned with ClustalW 2.1 [16] (BLOSM62 matrix) vs. *Aquifex aeolicus* LeuT and *Drosophila* DAT transporters which possess explicit structural annotations. Sequences that displayed apparent significant losses of functional domains were eliminated from the alignment, assuming that they may represent pseudogenes or have incorrect predictions of their ORFs. Amino and carboxyl termini that represented most divergent regions were trimmed in the alignment used for the tree (Fig 1). The tree was built by the FastTree 2.1.5 software [17, 18] (http://www.microbesonline.org/fasttree/) and inferred approximately-maximum-likelihood phylogenetic trees of the protein alignment. The reliability of each split in the tree estimated by FastTree used the Shimodaira-Hasegawa test on the three alternate topologies (NNIs) around that split. We used default 1,000 resamples setting in order to estimate local support values.

### Heterologous expression in *X. laevis* oocytes

The cRNA for the *C*. *capillata* transporter (*Cc*SLC6) used for oocyte injections was generated by *in vitro* transcription of a NotI-linearized pXOOM-*Cc*SLC6 plasmid with T7 polymerase using a mMessage mMachine ULTRA transcription kit (Invitrogen, Thermo Fisher Scientific). Surgically isolated and collagenase-treated stage V-VI *Xenopus laevis* oocytes (Nasco, Fort Atkinson, WI) were injected with approximatively 40 ng of *Cc*SLC6 cRNA and incubated for 3–7 days at 17°C in a sterile oocyte medium (98.0 mM NaCl; 2.0 mM KCl; 1.0 mM MgCl; 1.8 mM CaCl; 2.5 mM Na pyruvate; 100 units/ml penicillin; 100 μg/ml streptomycin; 5% horse serum; 10 mM Hepes, pH 7.4).

### Electrophysiological recordings

Currents from *Cc*SLC6-cRNA injected oocytes were recorded in a 50 μl constant-flow chamber at the time of the application of each of the following substances at millimolar concentrations: β-aminoethanesulfonic acid (taurine), β-alanine, *γ*-Aminobutyric acid (GABA), *5-hydroxytryptophan (*5-HTP), 5-hydroxytryptamine (5-HT), L-DOPA, dopamine, octopamine, epinephrine and norepinephrine as well as the L-form of the following amino acids, serine, threonine, asparagine, glutamine, tyrosine, aspartic acid, glutamic acid, arginine, lysine, and histidine. All substrates were tested at the concentration of 10 mM in the ringer solution except for less the soluble L-Tyr, which were tested at a concentration of 2.5 mM. The recordings were performed with an OC-725C Oocyte Clamp amplifier (Warner Instruments LLC, Hamden, CT, USA) and 2 M KCL-loaded glass microelectrodes with 0.5–1 MΩ resistance. Current and voltage signals were obtained and analyzed using DigiData 1322A ADC and pCLAMP software (Molecular Devices, Sunnyvale, CA, USA). The reference assay was based on the detection of substrate-induced current. Voltage clamp oocyte screening conditions for this assay were set at -50 mV [19, 20]. A total number of 76 oocytes were tested. The composition of solutions used to superfuse oocytes has been described earlier [9, 19, 20].

### *In situ* hybridization

The procedure was based on a protocol developed for mollusk Central Nervous Systems (CNS) [21] and adapted for *C*. *capillata* tissues. Riboprobes were prepared using a digoxigenin RNA labeling mix along with T7 and T3 RNA polymerases (Sigma-Aldrich, MO, USA). The full-length transporter cDNA from *C*. *capillata* cloned into pCR4-TOPO vector using HindIII and NotI restriction sites of the polylinker was linearized with PmeI or NotI enzymes. The digested fragments were purified using a PCR cleanup kit (Qiagen, Germantown, MD) and used as template for the generation of the antisense and sense probes by T7 or T3 RNA polymerase, respectively. RNA probes were then purified using the RNeasy MiniElute Cleanup Kit (Qiagen, Germantown, MD). The integrity of the probe was confirmed by electrophoresis on an ethidium bromide 1% agarose gel. Jellyfish were anesthetized in an isotonic solution of MgCl_2_ diluted 1:1 with sea water, then peri-rhopalial tissues were dissected and pinned down on Sylgard-184 plates (Ellsworth Adhesives, Germantown, WI). One set of peri-rhopalial tissues had their epithelia removed using the oxidative method [22]. Both intact and epithelial cell-free tissue samples were then fixed for 2 h at room temperature in 4% ParaFormaldehyde (PF) prepared in 0.1 M Phosphate Buffer (PB), pH 9.5 containing 0.42 M NaCl and 2 mM MgSO_4_ [PROTOCOL DOI: dx.doi.org/10.17504/protocols.io.3qngmve]. The alkaline fixation was reported to enhance signals in *in situ* hybridization procedures [23]. Following three washes in PBST (0.1 M Phosphate Buffer pH 7.5, 150 mM Sodium chloride, 0.1% Tween-20) of 5 min each, the fixed tissues were dehydrated in PBS/ethanol mixtures in the ratio of 3:1, 1:1, and 1:3 for 10 min each and kept overnight in 100% ethanol at -20°C. Tissues were then rehydrated by successive 10 min washes in PBS/ethanol mixtures in the ratio of 1:3, 1:1, and 3:1, and finally transferred in a capped 1.5 ml tube containing 800 μl PBST for a 5 min heat treatment at 95°C [24]. In order to prevent the glueing together of specimens during the heat treatment, it is important to re-suspend the samples at 20-sec interval within the two first minutes of heating, followed by additional shakings every 60 sec during the rest of the heat treatment. Samples were then chilled in an ice bath. This treatment considerably shrunk the peri-rhopalial tissues. Following two washes of 5 min each in PBST, tissues were treated 5 min in freshly prepared TEAA (0.1 M TriEthanolAmine pH 7.8 containing 0.25% Acetic acid), washed 2 times for 10 min in PBST and post-fixed for 20 min in 4% PF prepared in PBST, pH 7.5. After two consecutive washes of 10 min each in PBST, unspecific binding sites present on the tissue were saturated using heat-denatured molecular grade DNA from sperm of herring and cod that had a molecular weight distribution of 150–3000 bases. Each peri-rhopalial tissue was incubated at room temperature for 10 min in 75 μl of PBST containing 0.5 mg/ml of fish sperm DNA. Then 75 μl of the hybridization mixture (50% formamide, 5x Saline-Sodium Citrate buffer (SSC), 1 mg/ml sperm-DNA, 0.1% Tween 20) was added to each tissue sample and incubated at room temperature for 5 min. The solution was then replaced with 150 μl of hybridization mixture and tissues were incubated at room temperature for 10 min. Pre-hybridization in this solution was pursued at 45°C for 2 h. Hybridization was conducted at 40°C for 48 h using three different digoxigenin-labelled cRNA probe concentrations for both the anti-sense and sense probes (2250 ng/ml; 560 ng/ml; 56 ng/ml). Before use, probes were denatured in the hybridization mixture at 70°C for 10 min and then snap-cooled in an ice bath. Tissues were then washed two times in 50% formamide, 2x SSC, 0.1% Tween-20 at 45°C for 1 h, followed by four washes of 15 min in the same conditions. Tissues were equilibrated in PBST for 20 min at room temperature, then submerged in blocking buffer (10% Normal Goat Serum (NGS) in PBST) for 2 h with gentle shaking at 4°C, followed by an incubation with shaking at 4°C for 72 h with a DIGoxigenin antibody conjugated to Alkaline Phosphatase (AP-anti-DIG antibody) diluted 1:2000 in PBST containing 1% NGS. Tissues were rinsed at room temperature four times for 20 min each with PBST. After three washes of 10 min each in AP-buffer (100 mM Tris, pH 9.6; 100 mM NaCl; 10 mM Mg Cl_2_; 1 mM levamisole; 0.1% Tween-20), the labelling was developed in the dark in nitro blue tetrazolium chloride / 5-bromo-4-chloro-3-indolyl-phosphate (NBT/BCIP) solution (11681451001 Roche, Millipore Sigma). The colorimetric reaction was stopped by rinsing the tissues three times for 5 min with PBST containing 50 mM *Ethylene Diamine Tetraacetic Acid* (EDTA). Tissues were re-fixed in 4% PF for 30 min, then rinsed three times, 10 min each, in ethanol, and finally transferred in methyl salicylate. Tissues were mounted in a glycerol-based aqueous medium and digitally documented using Zeiss ApoTome.2 microscope equipped with CCD camera.

### RNA isolation, and RT-PCR using antisense strand-specific priming

Neurons of the Motor Nerve Net (MNN) were isolated from freshly isolated peri-rhopalial tissues after removal of the epithelia using the oxidative procedure [22]. Total RNAs were isolated from a pool of several preparations of motor neurons using RNAqueous-Micro Kit (Thermo Fisher Scientific, USA). RNAs were eluted twice in a volume of 10 μl water and the eluate was treated with TURBO DNase (Thermo Fisher Scientific/*Life Technologies*, USA). *RNAs were either purified on an* RNA Clean & Concentrator (Zymo Research Corp., Irvine, CA) or by lithium chloride precipitation. The quality of the RNA sample was assessed on an Agilent BioAnalyser. The presence of antisense strands expressed by the *Cc*SLC6 gene was identified using a modified RT-PCR procedure. First, total RNAs isolated from the neurons were reverse-transcribed using SuperScript III First-Strand Synthesis (Thermo Fisher Scientific, USA) along with a gene-specific primer annealing to the RNA antisense strand of the *C*. *capillata* transporter (nuc52-76: attgccaagacggagtccatcaagg). Five microliters of the cDNA product were then submitted to a PCR amplification in a volume of 50 μl using LA Taq DNA polymerase (Takara Bio USA, Inc.) along with the primer pair Forward 176 and Reverse 757 (For_176: TTTGTCAGAAGAATGGAGGAGGTGC; Rev_757: CCTTTCCGCTGGACTGGATACCTT), which were specific to the jellyfish transporter sequence. The conditions for the amplification were as follows: initial denaturation at 95°C, 7 min; 35 cycles of 30 sec at 60°C, and 90 sec at 72°C; and extension at 72°C for 10 min. Concurrently, controls that consisted of the same RNA sample used for the RT as well as a water control sample were both subjected to the PCR amplifications described above. One microliter of each of the PCR products was further submitted to a PCR amplification using the nested primers Forward 293 and Reverse 684 (For_293: CCCTCTTGGCATGGAAGGAAA; Rev_684: GAGTTTGGCATTGAGGCCGA) according the PCR conditions described above. All PCR products were electrophoresed on a 1% ethidium bromide agarose gel. A fragment in the range of 300 bp that was amplified in the experimental sample was cloned into a pGEM-T vector (Promega, USA) and the insert was sequenced.

### Immunocytochemistry

*Cc*SLC6 ovalbumine-conjugated peptides and anti-*Cc*SLC6 polyclonal antibodies were purchased from 21^st^ Century Biochemicals (Marlboro, MA). Rabbits were immunized with two peptides corresponding to regions located at the N-terminal end (MPEEMIEMDTPVQHDSC) and C-terminal end (CANYHPDPSWVDPSRRKSP) of the *Cc*SLC6 transporter. The serum from the second bleed was used in the immunolocalization experiment. Whole-mount immunochemistry was conducted on intact peri-rhopalial tissues fixed for 4 h at room temperature in 4% PF prepared in PB solution, pH 7.4 containing 2% NaCl. Then, tissues were extensively rinsed in PBS (0.9% NaCl) pH 7.4, and incubated in blocking solution (PBS, 0.1% Triton X-100, supplemented with 1% normal goat serum and 1% bovine serum albumin) for 2 h at room temperature. Peri-rhopalial tissues were incubated overnight at 4°C in presence of the whole immunized rabbit serum (protein concentration: 65μg/μl) diluted 1:100 in the blocking solution. The control experiment was conducted in the same conditions but tissues were incubated overnight with the rabbit’s pre-immune serum. Tissues were rinsed six times for 30 min each time in PBS after which they were treated with a secondary antibody FITC-conjugated goat anti rabbit IgG (ThermoFisher Scientific, Invitrogen, USA) diluted 1:500 in the blocking solution for 3 h at room temperature. After washing, the samples were mounted with anti-quench mounting medium Fluoromount-G (ThermoFisher Scientific, USA) and examined by confocal microscopy (Leica SP5).

### Immunofluorescence labelling of HEK 293 cells transfected with pXOOM-*Cc*SLC6

Human Embryonic Kidney HEK 293 cells [25] (ATCC, Manassas, VA) were maintained at 37°C in 5% CO_2_ in Dulbecco Modified Eagles Medium (DMEM) supplemented with 2mM L-glutamine, 10% Fetal Calf Serum (FCS), 100 I.U/ml penicillin, and 100 μg/ml of streptomycin. HEK-293 cells seeded on poly-l-lysined coverslips were transfected with the construct pXOOM-*Cc*SLC6 using Fugene 6 reagent (Promega, Madison, WI, USA). Following a 48-hour transfection, HEK 293 cells were fixed for 15 min at room temperature with a solution of 2% PF prepared in PBS-CM (PBS containing 0.1 mM CaCl_2_ and 1 mM MgCl_2_). The cells were then washed 10 times for 1 min each with PBS-CM and incubated for 1 h at room temperature with the blocking solution PBS-CM containing 1% Bovine Serum Albumin (BSA). The serum of the immunized rabbit containing the antibodies to *Cc*SLC6 was diluted 1:200 in the blocking solution and applied on the cells. Following an overnight incubation at 4°C with the primary antibody, the coverslips were washed five times for 1 min each after which the cells were incubated 2 h at room temperature in the secondary antibody Alexa Fluor 546 goat anti-rabbit IgG (Thermo Fisher Scientific, Invitrogen) diluted 1:500 in the blocking solution. Cells were then washed several times in PBS-CM and the coverslips were mounted on glass slides with 60% glycerol in PBS containing 1 mg/ml o-phenylenediamine HCL to minimize autofluorescence. The cells were viewed with a confocal microscope (Leica SP5).

## Results

### Molecular cloning of the first *C*. *capillata* transporter

As a result of a PCR performed on a *C*. *capillata* λgt22 phage library using a degenerate primer deduced from a protein sequence alignment of annotated SLC6, we isolated a cDNA sequence of 1953 bp, which encodes a protein of 651 amino acids with a deduced molecular mass of 72,707 Da (GenBank accession number MH737701 / protein_id QBP15011). The Kyte-Doolittle hydopathy analysis of the transporter predicted the presence of 12 transmembrane domains with intracellular C- / N- termini, and a large extracellular loop between the third and fourth transmembrane domains which is predicted to contain two N-glycosylation sites at position 173–176 (NLSD) and position 182–185 (NLTR) as well as a potential disulfide bridge (C153, C196, C197). These features are common among previously annotated and characterized SLC6 transporters [26].

### Phylogenetic position of the *Cc*SLC6 protein

The phylogenetic tree (Fig 1) was constructed to reveal the exact position of the new transporter among a subset of better characterized SLC6 transporters as well as SLC6 sequences retrieved from annotated genomes and transcriptomes of cnidarians and divergent organisms. The tree showed that the jellyfish transporter belongs to the NAT subfamily. The closest homologs of the *Cc*SLC6 sequence are cnidarian transporters of the starlet sea anemone (*N*. *vectensis)* A7RFF4 (62% and 70% for identity and BLSM62 homology, respectively), and a coral (*Sylophora pistillata*) A0A2B45J71 (64% and 74% for identity and BLSM62 homology, respectively). Human characterized homologs in the NAT cluster display about 55% of similarity with the *Cc*SLC6 sequence.

In consideration of the phylogenetic topology of the tree, we may predict that *Cc*SLC6 is functionally similar to NATs and therefore we will rename the transporter as *Cc*SLC6_NAT1. However, a more precise prediction of the function of *Cc*SLC6_NAT1 based on sequence homology with characterized NATs from the available phylogenetic information was impossible.

### *Cc*SLC6_NAT1 is predicted to be a Na^+^-dependent, Cl^—^independent transporter

**Fig 2:**
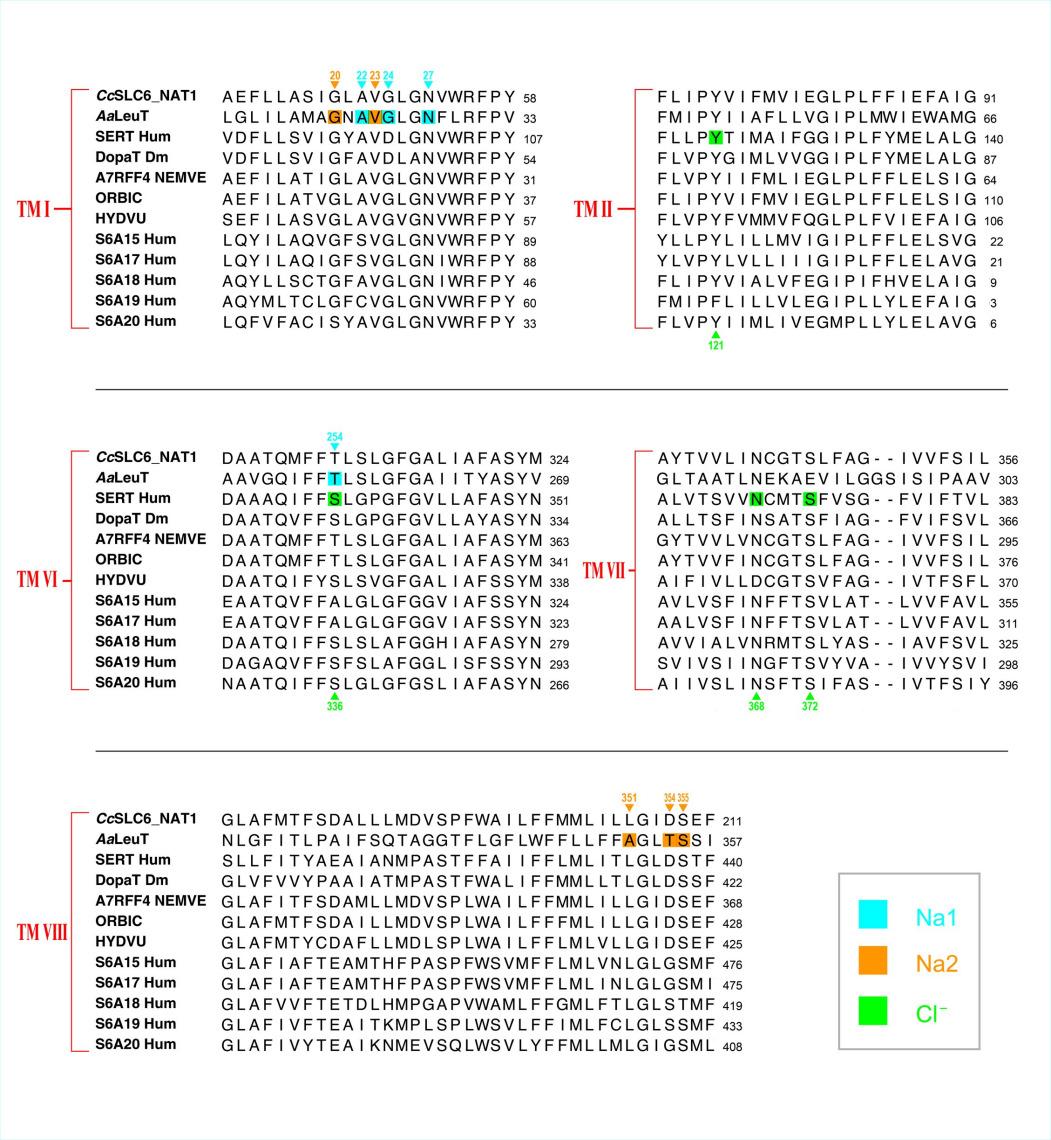
Sodium and chloride binding sites as identified in the bacterial *Aa*LeuT and the human serotonin (SERT) transporters. Transmembrane domains (TM I, II, VI, VII and VIII) that depict residues that interact with sodium 1 and 2 as well as with chloride are highlighted with the background colors blue for Na1 sites, orange for Na2 sites and green for Cl^-^ sites. Numbers located at the top of the alignment correspond to residue numbers of *Aa*LeuT known to interact with sodium 1 and 2, while residues numbered at the bottom of the alignment are those known to interact with chloride in SERT. The sites that coordinate the binding of sodium in the jellyfish sequence are strictly conserved for Na1 while only partly conserved for Na2. Although *Cc*SLC6 is likely to translocate its substrate in presence of sodium, the fact that position 309 in the jellyfish sequence is a threonine as it is in *Aa*LeuT may suggest that *Cc*SLC6 may not rely absolutely on Cl^-^ (see Results section for more details). The alignment is constituted of the *Cc*SLC6_NAT1 jellyfish sequence (Accession: MH737701 / protein ID: QBP15011) along with the following three sequences for which a crystalized form has been determined: *A*. *aeolicus Aa*LeuT (PDB ID 2A65), human serotonin transporter SERT Hum (NP_001036.1) and dopamine *Drosophila melanogaster* transporter DopaT Dm (NP_523763). The alignment also includes characterized human (Hum) as well as cnidarian sequences which are homologues of the *Cc*SLC6_NAT1 sequence in the phylogenetic tree (Fig 1). They are identified as follows: S6A15 Hum (NP_877499HS), S6A17 Hum (NP_001010898), S6A18 Hum (NP_872438.2), S6A19 Hum (NP_001003841), S6A20 Hum (NP_064593), A7RFF4 NEMVE for *Nematostella vectensis* (XP_001641770.1), ORBIC for *Orbicella faveolata* (XP_020611607), and T2M528 HYDVU for *Hydra vulgaris* (XP_012559838). The sequences were aligned using ClustalW tool in MacVector (v. 15.5.4).

SLC6 transporters translocate their substrates in presence of sodium ions and in most cases in conjunction with a chloride ion. When compared to the crystal structure of the homologous bacterial transporteur *Aa*LeuT sequence (Protein Data Bank ID 2A65) [27], residues that are involved in the binding of the sodium ions in *Aa*LeuT are strongly conserved in the *Cc*SLC6_NAT1 sequence (Fig 2). One site is strictly conserved (_*Cc*SLC6_NAT1_Na1: A47, G49, N52, T309 _/ *Aa*LeuT_Na1:A22, G24, N27, T254), while the sodium site that coordinates the binding of the second sodium (Na2) in *Aa*LeuT is only partly conserved in the *Cc*SLC6_NAT1 sequence. The jellyfish sequence possesses three residues (_*Cc*SLC6_NAT1_Na2: G45, V48, S409 /_*Aa*LeuT_Na2: G20, V23, S355) among five that are identical to the Na2 sites that reside in the binding pocket of the *Aa*LeuT sequence. As for the two other residues (_*Cc*SLC6_Na2: L405, D408), known to interact with Na2 in *Aa*LeuT (_*Aa*LeuT_Na2: A351, T354), only L405 of *Cc*SLC6_NAT1 is similar to the equivalent residues present in the *Aa*LeuT sequence. This slight difference in the binding site of Na2 of *Cc*SLC6_NAT1 is also observed in most neurotransmitter transporters (NTT). As far as the Na2 site is concerned, CcSLC6_NAT1 is more similar to NTT than to its counterparts of the NAT. However, it is still not clear to what extent the Na2 site is used in NTT during substrate translocation [27, 28]. Based on these observations, *Cc*SLC6 is probably using at least one sodium ion during translocation of its substrate. The same however cannot be said with regard to the chloride ion.

Contrary to what is observed in eukaryotes, transport of substrate mediated by SLC6 proteins in prokaryotes is chloride independent [29]. However, SLC6 transporters’ function requires chloride in most cases and the residues involved in the binding of this ion have been mapped. Those chloride binding sites typically found in mammalian neurotransmitter transporters are conserved in *Cc*SLC6_NAT1 (*Cc*SLC6_NAT1: Y72, N341, S354, compared to serotonin transporter SERT, NP_001036.1: Y121, N368, S372) [30, 31]. However, the residue S336 known to coordinate the Cl^-^ ion in SERT, which is conserved in many other members of the NTT and NAT families, is replaced by a threonine in *Cc*SLC6_NAT1 (T309), a residue that is also present in the *Aa*LeuT sequence at the corresponding position (T254). In fact, in prokaryotic sequences, this residue is believed to interact directly with a sodium ion, and together with residue _*Aa*LeuT_E290, _*Aa*LeuT_T254, would probably prevent the binding of chloride. In light of these observations, *Cc*SLC6_NAT1 may not rely absolutely on chloride ions in order to translocate its substrates. While transport in S6A18 Hum and S6A20 Hum is dependent on chloride, albeit not always in a strict fashion, not all NAT transporters of the B^0^ group rely on extracellular chloride to transport their substrate. For instance, Cl^—^independent B^0^ transporters such as S6A15 Hum, S6A17 Hum and S6A19 Hum, each display a non-conserved amino acid among the four residues known to coordinate the chloride ion [32, 33, 34], while both S6A18 Hum and S6A20 Hum, which interact with chloride during transport, have all four of their amino acids identical to the ones identified as chloride binding sites in SERT [35, 36]. When one compare Na^+^ and Cl^-^ binding sites conserved in *Cc*SLC6_NAT1 with other cnidarian sequences, NEMVE and ORBIC which share the highest homology with the jellyfish sequence display an identical set of residues presumed to be involved in ionic interactions. Conservation of the above sites among cnidarians decreases as the phylogenetic distance increases, which suggests that dependence on ions during transport of substrates for these transporters may vary considerably across the phylum.

### *Cc*SLC6_NAT1 expression in the motor nerve net of *C*. *capillata*

**Fig 3:**
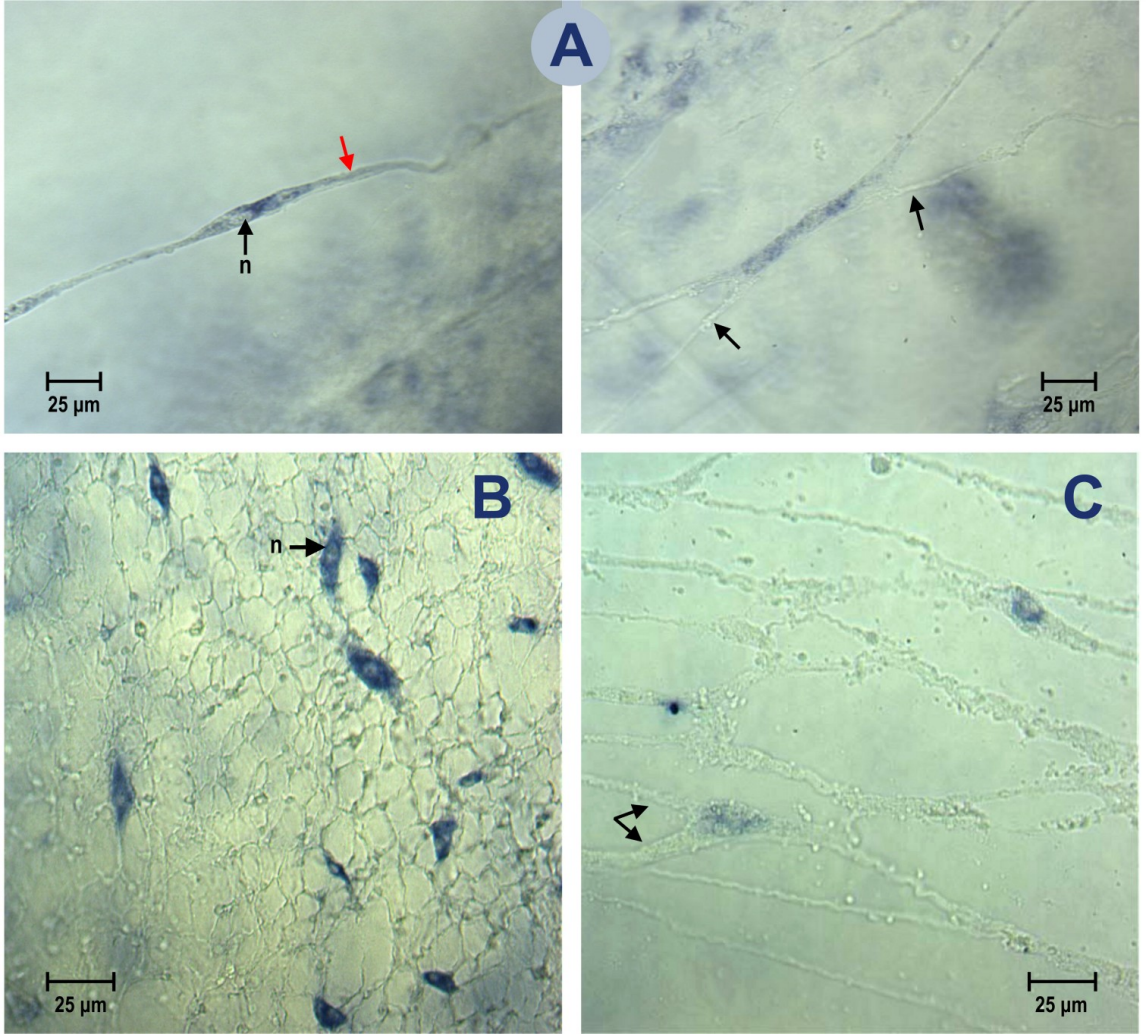
Distribution of the *Cc*SLC6-NAT1 transcripts in the MNN neurons of the peri-rhopalial tissue. *in situ* hybridization experiments were performed on whole-mount peri-rhopalial tissue using digoxigenin-labelled cRNA probes transcribed from the full-length cDNA of *Cc*SLC6_NAT1. (A) Epithelial cell-free preparations show two neurons (left and right panels) of the MNN attached to a transparent mesoglea. The purple precipitate in the cells highlights zones where the transporter transcripts are expressed. The transcripts are predominantly present in the cytosol of the neuronal somata, in the periphery of the nuclei (n), and occasional labeling is also observed in the processes (red arrowhead in left panel). Two arrows in right panel of (A) shows the process of one neuron that forms an axosomatic contact with the labelled neuron. (B) An *in situ* hybridization experiment conducted on intact peri-rhopalial tissue. Labelled somata of the neurons are seen through the transparent epithelial layer. No labelling was observed in epithelial cells. (C) The labeling obtained in the control experiment using the sense probe suggests that the transporter gene may undergo bidirectional transcription. Double arrowheads indicate two processes that emerged from one neuronal soma, while a third process lay at the opposite pole of the soma, typical of tripolar nerve cells.

*In situ* hybridization experiments were conducted on intact as well as epithelial cell-free peri-rhopalial tissues. In both preparations, hybridization experiments using the DIG-labelled anti-sense probe showed that the transporter’s transcripts were localized in the cytosol of neuronal somata in periphery of the nuclei (see epithelium-free peri-rhopalial preparation: Fig 3A, left and right panels, and intact peri-rhopalial preparation, Fig 3B). Labeling that was occasionally present in the neuron processes was too faint to be seen through the epithelial layer of the intact peri-rhopalial tissue (Fig 3B). Three probe concentrations were tested (56 ng/ml; 560 ng/ml; 2250 ng/ml), however only the results obtained with the highest probe concentration are shown as this concentration produced the most robust labelling. Nonetheless, because the *in situ* hybridization results obtained with the control sense probe labelled the neuronal somata of the motor neurons in the same fashion as the anti-sense probe, the experiment was repeated using an additional control experiment in order to assess the presence of any non-specific staining that could have been generated by the experimental procedure. This control experiment, conducted on intact peri-rhopalial tissues, used a sense cRNA probe synthetized from the full-length cDNA sequence encoding an uncharacterized G-protein coupled receptor isolated from a *C*. *capillata*. Because the distribution of labelling produced by the anti-sense GPCR probe was different from the one obtained with the probe specific to the *Cc*SLC6_NAT1 transcripts, we are confident that the *in situ* hybridization results obtained in this present study are reliable. A related point to consider is that, contrary to the *Cc*SLC6_NAT1 sense probe, the GPCR sense probe behaved as a genuine control as it did not label any target in the peri-rhopalial tissue. Because the distribution of the labeling obtained with the GPCR probe was not in the MNN neurons further supports the fact that the labelling obtained with the *Cc*SLC6 control probe was not a false positive but rather a consequence of the expression of the sense transcripts for this gene.

**Fig 4:**
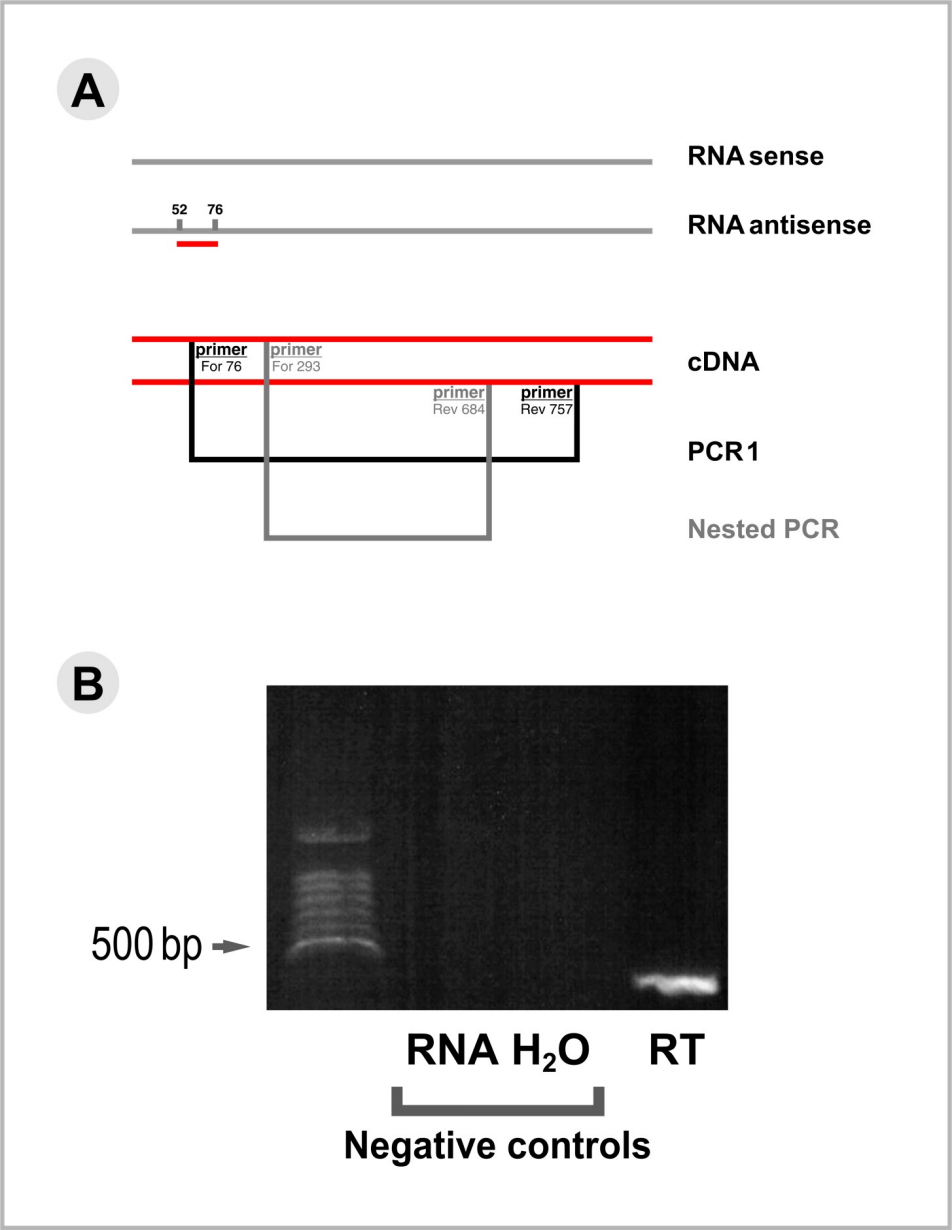
Strategy used to detect the expression of antisense transcripts of the *Cc*SLC6_NAT1 gene. (A) A DNA primer (primer 52–76) complementary to the sequence of the antisense transcript was used in a reverse transcription procedure on total RNAs isolated from neurons of the peri-rhopalial tissue. Complementary DNAs obtained by this modified reverse transcription were then subjected to a PCR amplification (PCR1) using the primer pair For_76 and Rev_756. As no amplification product from PCR1 was visible on agarose gel, one microliter of this product was further subjected to a nested PCR using primer pair For_293 and Rev_684, and the same amplification conditions described for PCR1 (see Material & Methods). Numbers used to identify PCR primers indicate the nucleotide positions on the *Cc*SLC6 cDNA to which the 5’ terminal base of the primer would anneal to. (B) Lanes of the agarose gel are identified according to the type of template (genuine or mock) used in PCR1 amplifications. Lane RNA: DNAse-treated RNA isolated from the MNN; Lane H_2_O (water); Lane RT: reverse transcription conducted on DNAse-treated RNA isolated from the MNN. The product of the nested PCR generated a band of the expected size (392 bp, lane RT on the ethidium bromide 1% agarose gel). The identity of the band was confirmed by sequencing. The negative control experiment consisted in an amplification (PCR1) of the same RNA sample, which came from the same pool of RNA used for the reverse transcription (lane RT). One microliter of this PCR1 product was then subjected to a nested PCR. Absence of amplification of this control sample suggested that genomic DNA was effectively eliminated from the total RNA preparation following the DNAse treatment. Therefore, the amplification of a fragment of the expected size in the experimental sample (lane RT) was the result of the first-strand cDNA synthesis of the antisense template during the reverse transcription procedure. A second control (lane H_2_O) consisting of a mock amplification using water (lane H_2_O) assessed any potential contamination of reagents used during PCR amplifications.

We then sought to explain the labelling of the neurons with this sense control probe by demonstrating that naturally occurring antisense transcription occurs for the *Cc*SLC6_NAT1 gene. In this procedure, it is important to remove any genomic DNA contaminant from the total RNA preparation isolated from the motor neurons. The DNAse-treated total RNA preparation was presumed to contain the antisense transcripts for the *Cc*SLC6_NAT1 gene, so we used a specific DNA primer that annealed to the antisense transcript in order to generate a complementary cDNAs during the reverse-transcription procedure (Fig 4A). Complementary DNAs of the antisense transcripts were then PCR amplified using a pair of primers specific to the *Cc*SLC6_NAT1 cDNA. Separation of the PCR product revealed a band in the range of the expected molecular weight (Fig 4B, lane RT). Sequencing of this band showed that the amplified sequence corresponded to the targeted region of the *Cc*SLC6_NAT1 cDNA. The control for this experiment consisted in a PCR amplification performed on the DNAse-treated RNA preparation. As the negative control (Fig 4B, lane RNA) did not generate a product, we are confident that the product obtained from the PCR amplification of the reverse-transcribed DNAse-treated RNAs is evidence of the presence of antisense transcripts, and not a consequence of genomic contamination of the total RNA preparation used for this experiment.

**Fig 5:**
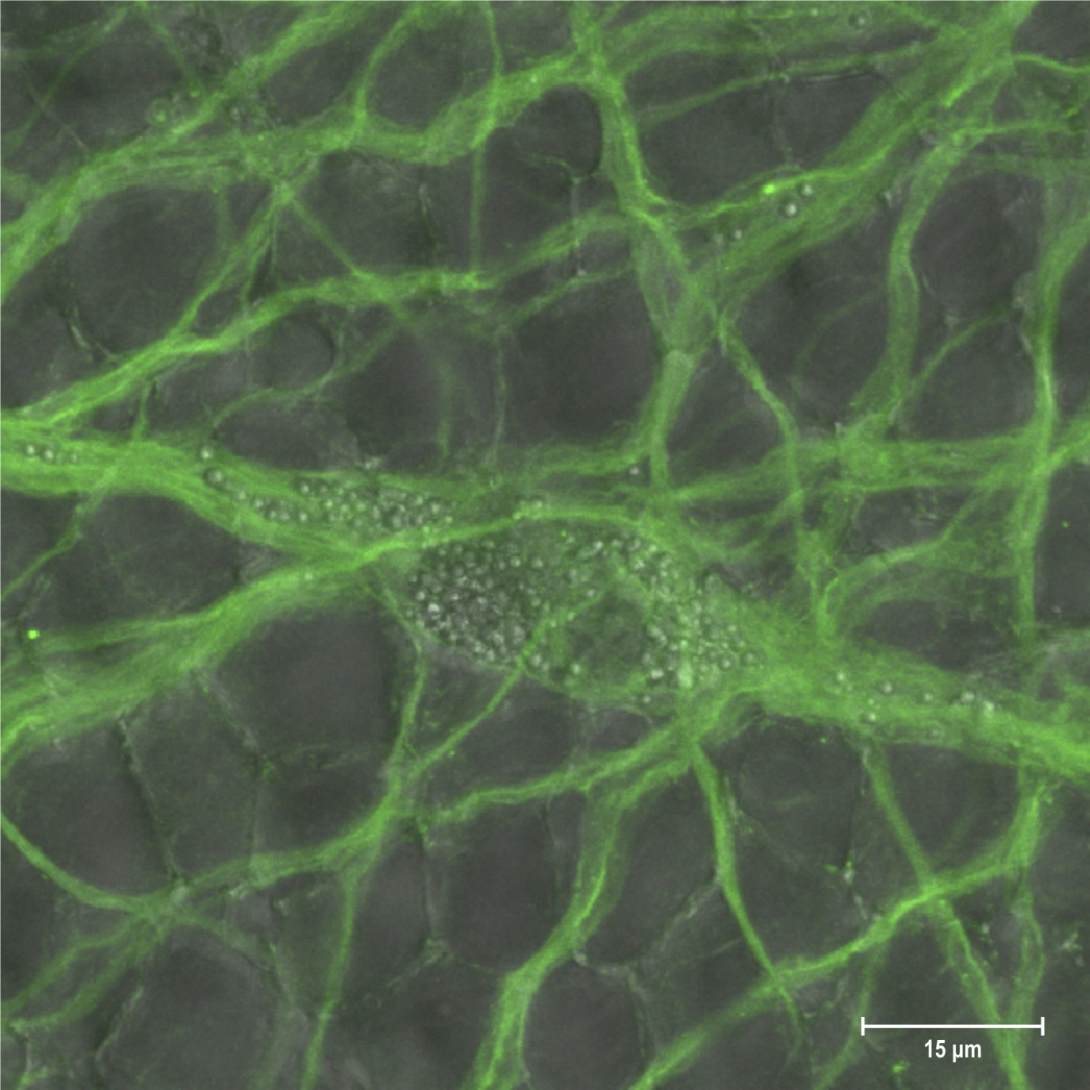
Cellular localization of the *Cc*SLC6_NAT1 protein in the peri-rhopalial tissue. Immunohistochemistry conducted on intact peri-rhopalial tissue using the polyclonal *Cc*SLC6_NAT1 antibodies (green). The MNN neurons expressed the CcSLC6_NAT1 transporter robustly on the surface of the cytoplasmic membrane.

Immunochemistry performed on intact peri-rhopalial tissues confirmed the results obtained with *in situ* hybridization experiments. The whole serum from a rabbit immunized with two peptides deduced from the N-terminal and C-terminal extremities of the *Cc*SLC6_NAT1 transporter generated a robust signal in the motor neurons of the peri-rhopalial tissue (Fig 5). As the two antigenic peptides were deduced from regions that are not conserved among members of the SLC6 family, the antibody is less likely to label other SLC6 transporters that might be expressed in the MNN, although we cannot exclude the possibility of other proteins present in the jellyfish MNN that would also be recognized by the antibody. The labelling revealed that the *Cc*SLC6_NAT1 protein expression is widespread in the plasma membrane of both somata and neurites of the motor neurons in this region. No labelling was observed in the control experiment which was performed using the rabbit pre-immunized serum. The labelling obtained with the *Cc*SLC6_NAT1 antibody was not restricted to the motor neurons. Preliminary results suggested that other neuronal types may be immunoreactive to this antibody.

**Fig 6:**
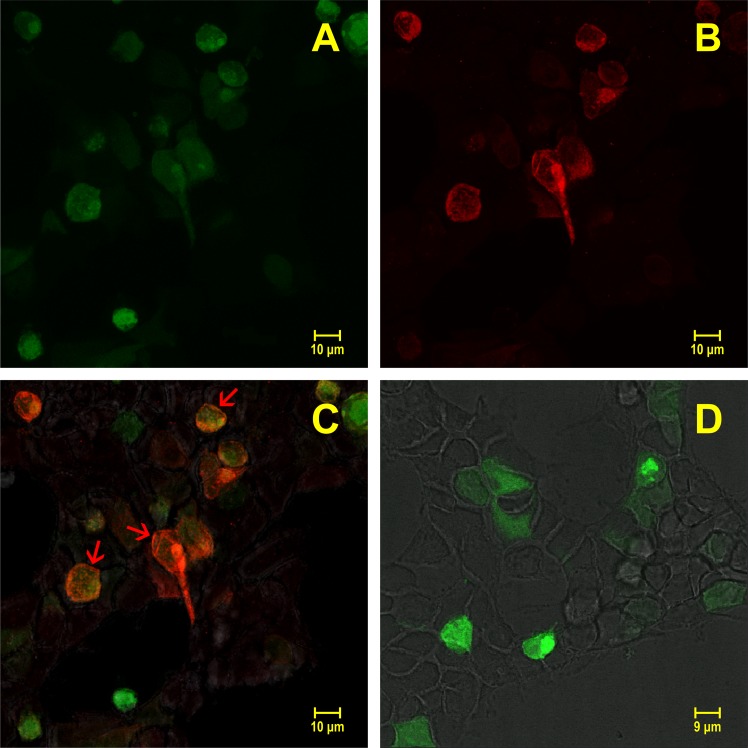
Heterologous expression of the *Cc*SLC6_NAT1 protein showed that the antibody recognizes the *C*. *capillata* transporter. The expression vector contained the GFP gene under the control of a promoter independent from the transporter’s promoter. (A) All cells that were transfected with the recombinant vector expressed GFP (green) in the cytosol of the HEK-293 cells. (B) GFP-expressing cells also expressed the transporter as revealed by the immunofluorescence experiment using the *Cc*SLC6_NAT1 antibody (red). (C) Merged images (A) and (B): all cells expressing GFP (green) also expressed the *Cc*SLC6_NAT1 transporter (red). Expression of the transporter was targeted at the plasma membrane (arrowheads). (D) Control (merged image). Immunocytochemistry experiment was also performed on transfected cells using the serum of the rabbit prior to immunization. The rabbit pre-serum did not label the transfected cells (absence of red fluorescence).

The ability of the antiserum to recognize the *Cc*SLC6_NAT1 transporter was verified on HEK 293 cells expressing transiently the transporter. In this experiment, the recombinant vector pXOOM contained the cDNAs of GFP and *Cc*SLC6_NAT1, both under the control of different promoters. Consequently, all transfected cells that produced green fluorescence (Fig 6A) were also expressing the *Cc*SLC6_NAT1 protein (Fig 6B). Immunofluorescence experiments using the serum of the immunized rabbit showed that the *Cc*SLC6_NAT1 protein was expressed at the level of the cytoplasmic membrane of the HEK-293 cells (Fig 6B and 6C). Such immunopositive cells were not observed in transfected cells treated with the rabbit pre-serum (Fig 6D).

Because the jellyfish transporter’s expression in a heterologous system was targeted to the plasma membrane of HEK 293 cells, we undertook to test a variety of substrates on *Xenopus laevis* oocytes injected with *Cc*SLC6_NAT1 cRNA. As none of the amino acids tested were transported in this heterologous system, it is possible that uptake by *Cc*SLC6_NAT1 requires the presence of an associated protein for membrane expression in oocytes [37, 38]. Alternatively, it is possible that an uncommon amino acid or an unidentified substrate, not tested in the uptake assay, is transported by the jellyfish transporter. Furthermore, this unknown substrate is expected to be translocated by cnidarian sequence homologs that cluster with the jellyfish transporter in the phylogenetic tree because they share identical residues at substrate-binding sites (S1 Fig).

## Discussion

We have isolated the first SLC6 sequence from the jellyfish *C*. *capillata* and we have shown by *in situ* hybridization and immunohistochemistry experiments that the transporter is localized in the MNN neurons of the peri-rhopalial tissue. The position of the new transporter sequence in a phylogenetic tree of members of the SLC6 family indicates that the new SLC6 sequence belongs to a cluster of cnidarian sequences likely ancestral to the nutrient amino acid transporter (NAT) subfamily. The *Cc*SLC6_NAT1 is the first identified candidate transporter that may play a role in neuronal homeostasis in a well-studied subset of neurons in a scyphozoan.

In *C*. *capillata* as well as other scyphozoan jellyfish, swimming movements are coordinated by neurons of the MNN (MNN–also named the Giant Fiber Nerve Net, GFNN by Horridge [39]) [40]. The MNN consists of a flat, single-layer sheet of relatively large bi-polar neurons along with occasionally tri-polar nerve cells, that are attached to an acellular mesoglea on the subumbrellar side of the bell [41]. Within this spatially diffused network, chemical neurotransmission proceeds by means of atypical synaptic contact, that is—vesicles are observed at both pre- and post-synaptic terminals [42, 43]. This structural characteristic accounts for the bi-directional propagation of the neuronal electrical activity within the MNN [44, 45]. The electrical signal relayed by the neurons of the MNN is principally generated in sensory organs distributed around the margin of the bell, and which are named rhopalia [43, 46]. These rhopalial structures contain pace-making as well as sensory cells that produce electrical activities that will ultimately trigger or modulate muscle contractions *via* synapses between neurons of the MNN and processes of muscle cells [47, 43]. The simplicity of the organization of this neuronal network allows for the study of neurons in their natural environment. A triangular section of the subumbrellar region of the bell along with its rhopalium and the MNN can be isolated ([45], see Fig 2). In this preparation, the area that accommodates the MNN is identified as the peri-rhopalial tissue. Provided that the single-layer subumbrellar epithelium is removed [22], the exposed neurons of the MNN can then be studied using electrophysiology with their original connections that remain linked to the various inputs generated in the rhopalium [48, 49, 44]. Intracellular recordings have shown that transmitters are released by action potentials [50]. Subsequently, functional studies conducted on the motor neurons of the MNN identified nonstandard substances as neurotransmitters, while none of the conventional transmitters tested on the neurons of the MNN were able to induce a response [51]. The substances able to evoke a robust depolarizing response in the neurons of the MNN were the beta amino acids taurine and β-alanine which are small amino acids structurally related to *γ*-aminobutyric acid (GABA) and glycine. These substances were released by depolarization of the MNN neurons along with aspartate, GABA, glutamate, glycine and possibly also some alanine and serine [51]. Interestingly, taurine was localized by immunochemistry in the neurons of the MNN [52], although one should mention that Satterlie & Eichinger [53] were not able to replicate this experiment in the MNN of *C*. *capillata* or, indeed, in any other scyphozoan jellyfish they tested.

Although taurine and β-alanine are presumed to be neurotransmitters in the vertebrate nervous system, a receptor sequence for taurine has not yet been isolated and β-alanine receptor sequences isolated in vertebrates ([54] contra [55]) do not display obvious sequence similarity with any cnidarian sequence available in databases. Conversely transport of taurine and β-alanine in the nervous system is prominent and the role that those substances play in all tissues is not restricted to neurotransmitter functions. While a few taurine transporter sequences were isolated from vertebrates and invertebrates, transporter- and receptor-mediated effects of both taurine and β-alanine also occur as a result of binding to receptors and transporters of both GABA and glycine (see reviews: [56, 55]). Inversely, taurine transporters are also sensitive to GABA, β-alanine as well as other amino acids [57]. The translocation of multiple substrates by those transporters may have its evolutionary roots in duplication events of a common ancestral transporter gene before the separation of the protostome and deuterostome lineages. Kinjo and colleagues [58] suggested that an initial duplication event, before the appearance of bilateral animals, resulted in the emergence of a creatine transporter and a GABA transporter. Their analysis proposed that taurine transporters from protostome animals have a different phylogenetic history than the taurine transporters of deuterostome animals. Even if those two groups of taurine transporters are functionally similar, taurine transporters in deuterostome animals are phylogenetically closer to the GABA transporters while taurine transporters in protostome animals form a clade with creatine transporters in a phylogenetic tree [58]. Interestingly, the creatine transporter has not been found at all in protostome animals which may imply that transport of creatine is performed by taurine transporters in this group of invertebrate animals. With this information at hand, searching for a taurine transporter in any representative of an ancient phylum that pre-date the appearance of explicit bilateral animals, such as cnidarians, using sequence information available from protostome or deuterostome animals could be misleading. If there were a taurine transporter in cnidarians, it may indeed be more similar to a GABA (of type GAT-1) or a creatinine transporter than any of the taurine transporters currently characterized in protostome and deuterostome animals.

Several initial attempts to isolate receptors and transporters of transmitters, including those of taurine, from partial transcriptomes of MNN neurons of the lion’s mane jellyfish failed in our hands. So we cast the net wider and searched for membrane proteins that were most prevalent and well-conserved across the phylogeny. Members of the SLC6 family were good candidates because this family is more diverse and ancient, with the NAT subfamily being the most basal, whereby a wide range of amino acids are transported. Some of these amino acids play crucial roles in neuronal homeostasis and have the potential to be either neurotransmitters or their precursors. This approach allowed for the isolation of a *Cc*SLC6 sequence that displayed similarities with transporters of the NAT subfamily. Subsequent to the isolation of the *Cc*SLC6_NAT1 sequence, transcriptomes of *Cyanea* species from the tentacles [59, 60] and the peri-rhopalial tissue [61] have been made available. Analyses of those transcriptomes should provide access to valuable sequence information to further our understanding of the nervous system of *C*. *capillata*.

The SLC6 family forms two major clusters on the tree, which are the Neurotransmitter transporter (NTT) and the Nutrient Amino acid Transporter (NAT) subfamilies. It is known that NTT genes underwent splitting in the course of early metazoan evolution and evolved under the ultimate pressure of conserving their specificity to a particular type of neurotransmitter [9, 13]. NTT members are made up of orthologous sequences (substrate-specific branches) where GABA transporters, catecholamine (dopamine, norepinephrine, and octopamine) transporters, indoleamine (serotonin) transporters, and glycine transporters form specific clusters, each constituted of transporters from different species. Hence, one can predict the substrate specificity of a new invertebrate transporter by the affiliation of this transporter with a particular cluster of the NTT subfamily [9]. In contrast, Nutrient Amino acid Transporter (NAT) sequences diverged under the evolutionary pressure of feeding, nutrient availability, and other environmental adaptations [11]. As a result, the NAT group evolved under rapid gene duplication events along with adaptive selections of transporters to new substrates. For this reason, members of the NAT subfamily tend to form paralogous clusters (species-specific). The conclusion that can be made based on this phylogenetic analysis is that the jellyfish transporter is not a member of the NTT subfamily, and accordingly does not transport either taurine or GABA. Indeed, the new sequence belongs to an ancestral group of the NAT subfamily and is part of a cluster of NAT cnidarian sequences, which possibly share similar substrate selectivity profiles (S1 Fig). On the phylogenetic tree, the best studied transporters of the NAT subfamily are characterized by their ability to translocate a wide spectrum of neutral amino acids [62]. On the tree they form two distinct groups: one group comprises human transporters B^0^AT2 /SLC6A15 (S6A15), NTT4 /XT1/SLC6A17 (S6A17) and the orphan SLC6A16 (SA16), whereas the second group of the system B^0^ includes B^0^AT1/SLC6A19 (S6A19), B^0^AT3/SLC6A18 (S6A18) and IMINO/SLC6A20 (S6A20) transporters. While transporters of the latter group are prevalent in epithelial membranes of the proximal kidney tubule [63] and gastrointestinal tract [35, 64], members of the first group are predominantly expressed in neuronal tissues as revealed by RT-PCR (v7-3/ B0AT2/SLC6A15 [65]), *in situ* hybridization (v7-3/B0AT2/SLC6A15 [66] and Rxt1/NTT4/SLC6A17 [67]), and immunohistochemistry (v7-3/B0AT2/SLC6A15 [58]; Rxt1/NTT4/SLC6A17 [68, 69]). The physiological role of those neuronal transporters is not well understood [70].

The localization study of *Cc*SLC6_NAT1 performed in the peri-rhopalial tissue showed a robust transcription of the *Cc*SLC6_NAT1 gene as well as its translated product in the neurons of the MNN. *Cc*SLC6_NAT1 transcripts were abundant in the cytosol in periphery of the nucleus. Additionally, the presence of transcripts in the fine neuronal processes was suggested by occasional faint labeling, a result that is consistent with axoplasmic transport of transcripts [71]. However, an *in situ* hybridization signal was also observed using the sense control probe in the cytosol of MNN neurons. Sense probes that reveal the presence of non-coding RNAs provide valuable information on gene expression. In fact, the conservation of non-coding RNAs in bacteria, yeast and plants as well as in invertebrate and vertebrate animals, is evidence of the importance of these transcripts in evolution [72]. Many genes produce transcripts from both DNA strands [73] and while the transcripts from the template strand are translated, complementary non-coding transcripts play important regulatory roles in cells [74] and in organs such as those involved in brain development [75]. Among those transcripts that do not code for a protein, the long non-coding RNA (lncRNA) is a class of non-coding RNAs that are more than 200-nucleotide-long. Based on the length of the *Cc*SLC6_NAT1 non-coding transcript region examined in the RNA sample of the MNN (nucleotide 52 to 757), we suggest that a lncRNA is expressed by the *Cc*SLC6_NAT1 gene. The *in situ* hybridization results were supported by immunochemistry experiments performed with the serum of a rabbit immunized with two peptides deduced from regions of *Cc*SLC6_NAT1 that are known to be the least conserved among members of the SLC6 family. The jellyfish transporter was widely expressed in the plasma membrane of the neurons of the MNN at the level of both soma and neurites.

Prior studies on amino acid transport in cnidarians concerned uptake of dissolved organic materials in the ocean. Those studies conducted on whole sea anemones and isolated cells from their dissociated tentacles suggested that there were three [76] and nine [77] separate transport systems for amino acids including systems that transported neutral amino acids with broad specificity. However, transporters involved in these systems have been neither molecularly isolated nor localized in cnidarians. Furthermore, it is to be expected that dissolved organic material in the ocean represents only a fraction of all substrates transported in cnidarians. Bioprospecting has revealed that marine animals synthesize a plethora of compounds absent in vertebrate and other terrestrial animals, and many remain yet to be discovered [78]. For this reason, functional characterization of transporters and receptors in cnidarians may also require the isolation and identification of new molecules.

In contrast to vertebrate and some invertebrate animals such as insects, cnidarians do not have a neurovascular unit or a hemolymph-brain barrier that distributes nutrients to neurons [79]. However, in cnidarians, the mesoglea is a rich source of amino acids [80] that can be released under the action of matrix metalloproteases [81]. How neurons in cnidarians maintain homeostasis in the absence of endothelial and glial cells will require investigating transport systems of these nutritious metabolites in the neurons themselves. The extensive distribution of *Cc*SLC6_NAT1 in the MNN neurons and the likelihood that at least one amino acid is translocated by the transporter suggest that *Cc*SLC6_NAT1 might be found to play a predominant role in neuronal nutrition.

Because our efforts in identifying substrates transported by the *Cc*SLC6_NAT1 in a heterologous system were not successful, in a future study we intend to determine the putative substrates used by CcSLC6_NAT1 using *in silico* modeling of the transporter’s binding site. As a preliminary immunolabeling study suggested that the *Cc*SLC6_NAT1 antibody may label other neuronal types in the jellyfish, we will also assess the overall distribution of the transporter in the entire animal. We are indeed interested to know whether this transporter could be expressed ubiquitously in all neuronal types in this jellyfish. If such were to be the case, it would then mean that *Cc*SLC6_NAT1 plays a fundamental role in neuronal function in the Lion’s mane jellyfish, and possibly, in other scyphozoans.

## Supporting information

S1 Fig Substrate binding sites of selected SLC6_NAT transporters. Sites interacting with substrates were identified from sequence alignments guided by phylogenetic information and 3D structural alignments of a set of well characterized transporters. The burgundy rectangles with patterned ends inserted on top of the alignment indicate transmembrane domains (TMDs) distribution. The inferences of amino acid binding sites are based on published occlusion state of *Aa*LeuT/3F3D (Yamashita et al., 2005). Only amino acids that exhibit disagreement with the consensus sequence are displayed on a background color; all other sites are shown on white background. The Ofa_NAT sequence corresponds to the *O*. *faveolata* ORBIC sequence in the tree (Fig 1). Although it is annotated as a GABA transporter in public database, the alignment shows that the major determinants known to be involved in the formation of the amino acid binding pocket among NAT are conserved in the ORBIC sequence.
(TIF)

S1 Data Trimmed alignment used to create the dendrogram of Fig 1. (TXT)
