# Supplementary figures and images for: A SLC6 transporter cloned from the lion's mane jellyfish (Cnidaria, Scyphozoa) is expressed in neurons
Source: PLoS One. 2019 Jun 24;14(6):e0218806. doi: 10.1371/journal.pone.0218806 (PMC6590891; doi:10.1371/journal.pone.0218806)

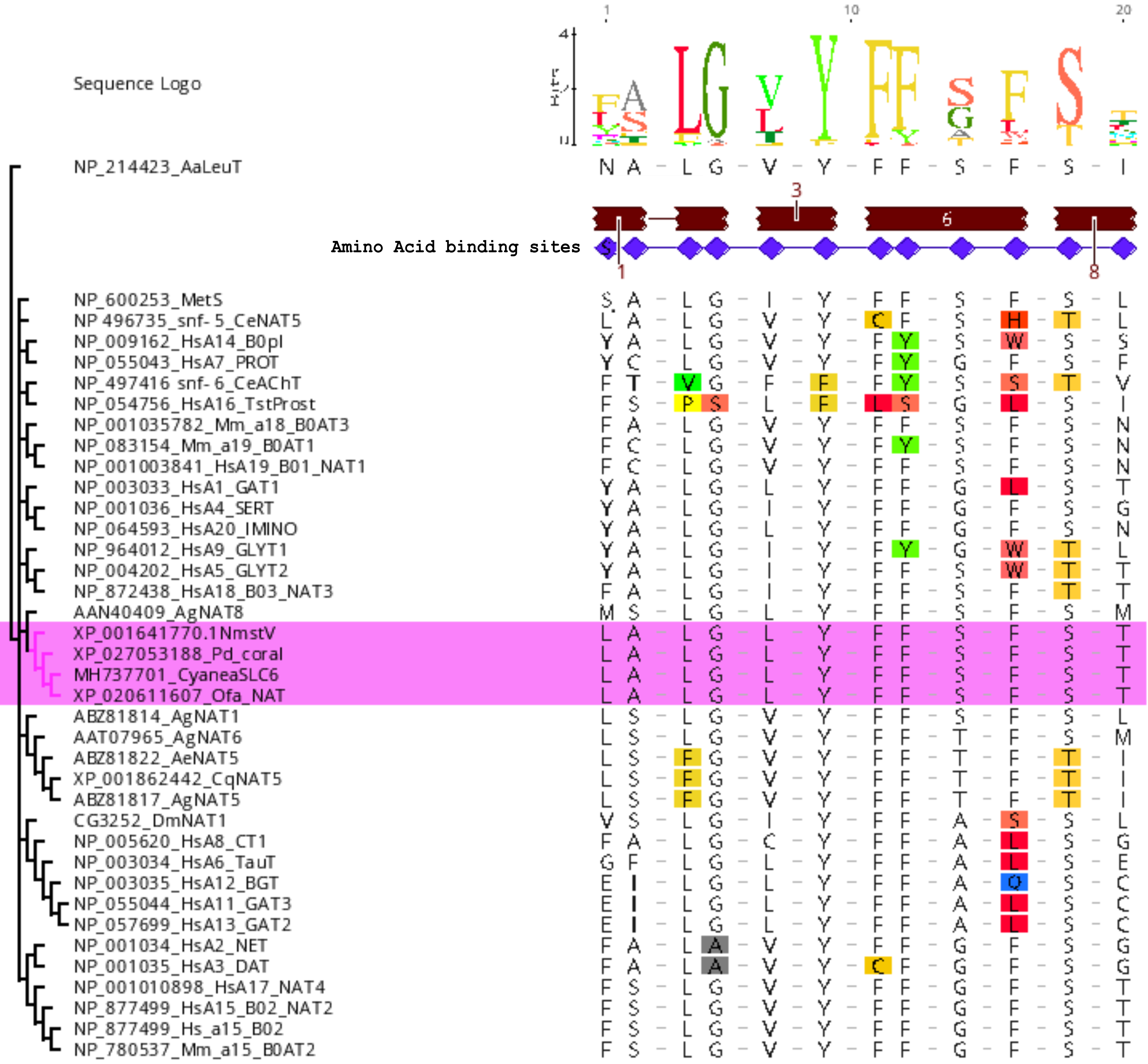

Supplement: S1 Fig — Sites interacting with substrates were identified from sequence alignments guided by phylogenetic information and 3D structural alignments of a set of well characterized transporters. The burgundy rectangles with patterned ends inserted on top of the alignment indicate transmembrane domains (TMDs) distribution. The inferences of amino acid binding sites are based on published occlusion state of AaLeuT/3F3D (Yamashita et al., 2005). Only amino acids that exhibit disagreement with the consensus sequence are displayed on a background color; all other sites are shown on white background. The Ofa_NAT sequence corresponds to the O. faveolata ORBIC sequence in the tree (Fig 1). Although it is annotated as a GABA transporter in public database, the alignment shows that the major determinants known to be involved in the formation of the amino acid binding pocket among NAT are conserved in the ORBIC sequence. (TIF) [file pone.0218806.s001.tif]
